# Supplementary material for: Evaluation of Polyacrylonitrile Nonwoven Mats and Silver–Gold Bimetallic Nanoparticle-Decorated Nonwoven Mats for Potential Promotion of Wound Healing In Vitro and In Vivo and Bone Growth In Vitro
Source: Polymers (Basel). 2021 Feb 9;13(4):516. doi: 10.3390/polym13040516 (PMC7915554; doi:10.3390/polym13040516)
Supplement: Supplementary file 1 [file polymers-13-00516-s001.pdf]

Supplementary materials

Article

Evaluation of Polyacrylonitrile Nonwoven Mats and Silver–Gold Bimetallic Nanoparticle–Decorated Nonwoven Mats for Promotion of Bone Growth and Wound Healing: *In Vitro* and *In Vivo* Studies

Meng-Yi Bai [1,2], Fang-Yu Ku [1], Jia-Fwu Shyu [3] Tomohiro Hayashi [4] and Chia-Chun Wu [5*]

1 *Graduate Institute of Biomedical Engineering, National Taiwan University of Science and Technology, Taipei 10607, Taiwan; email: mybai@mail.ntust.edu.tw*
2 *Adjunct Appointment to the Department of Biomedical Engineering, National Defense Medical Center, Taipei 11490, Taiwan.*
3 *Department of Biology and Anatomy, Tri-Service General Hospital, National Defense Medical Center, Taipei 11490, Taiwan; email: shyujeff@ndmctsgh.edu.tw.*
4 *Tokyo Institute of Technology, Department of Materials Science and Engineering, School of Materials and Chemical Technology, 4259 Nagatsuta-cho, Midori-ku, Yokohama, Kanagawa 226-8503, Japan.*
email: tomo@mac.titech.ac.jp
5 *Department of Orthopaedic, Tri-Service General Hospital, National Defense Medical Center, Taipei 11490, Taiwan; email: doc20281@gmail.com*

* Correspondence: *Dr. C. C. Wu, doc20281@gmail.com*

Received: date; Accepted: date; Published: date

Abstract: We prepared polyacrylonitrile (PAN) and urchin-like Ag–Au bimetallic or Ag nanoparticle–decorated PAN nonwoven mats using electrospinning and evaluated them *in vitro* and *in vivo* for promotion of bone ingrowth and wound healing and antibacterial effects on skin tissue. A facile, green, low-temperature protocol was developed to obtain these nonwoven mats. The sterilization rate of urchin-like Ag–Au bimetallic and Ag nanoparticle–decorated PAN nonwoven mats against *Staphylococcus aureus* was 96.81% ± 2.81% and 51.90% ± 9.07%, respectively, after 5-h treatment. In an *in vitro* cell model, these two mats did not show significant toxicity; cell viability of >80% was obtained within 5 h of treatment. *In vivo* animal model preclinical assessment showed that the urchin-like Ag–Au bimetallic nonwoven mat group showed good wound recovery because of sebaceous gland, hair follicle, and fat formation during skin tissue regeneration; increased neovascularization and compact collagen fibers were observed in the dermal layer, comparable to the findings for the control group. The mother substrate of the urchin-like Ag–Au bimetallic nanoparticle–decorated PAN nonwoven mats, that is, pure PAN nonwoven mats, was found to be an ideal scaffold for bone tissue engineering as osteoblast ingrowth from the top to the bottom of the membrane and proliferation inside the membrane were observed. The key genetic factor

Cbfa1 was identified as a key osteoblast differentiation regulator *in vivo*. Thus, electrospun membrane materials show potential for use as dual-functional biomaterials for bone regeneration and infection control and composite grafts for infectious bone and soft tissue defects.

Keywords: polyacrylonitrile; silver nanoparticles; electrospinning; Ag–Au bimetallic nonwoven mat; bone ingrowth; nonwoven mat; bone defect; soft tissue defect; urchin-like

Table S1 Magnusson and Kligman scale.

	Patch test reaction	Grading scale
·	No visible change	0
·	Discrete or patchy erythema	1
·	Moderate and confluent erythema	2
·	Intense erythema and swelling	3

Table S2 Score System of Skin Reaction.

Reaction	Primary Irritation Score
**Erythema and eschar formation**	
· No erythema	0
· Very slight erythema (barely perceptible)	1
· Well-defined erythema	2
· Moderate erythema	3
· Severe erythema (beet redness) to eschar formation preventing grading or erythema	4
**Edema formation**	
· No edema	0
· Very slight edema (barely perceptible)	1
· Well-defined edema (edges of area well-defined by definite raising)	2
· Moderate edema (raised approximately 1 mm)	3
· Severe edema (raised more than 1 mm and extending beyond exposure area)	4

Table S3 Score system of skin irritation evaluation.

Evaluations	Mean Score
· Non-irritant	0~0.4
· Slight irritant	0.5 ~ 1.9
· Moderately irritant	2.0 ~ 4.9
· Severely irritant	5.0 ~ 8.0

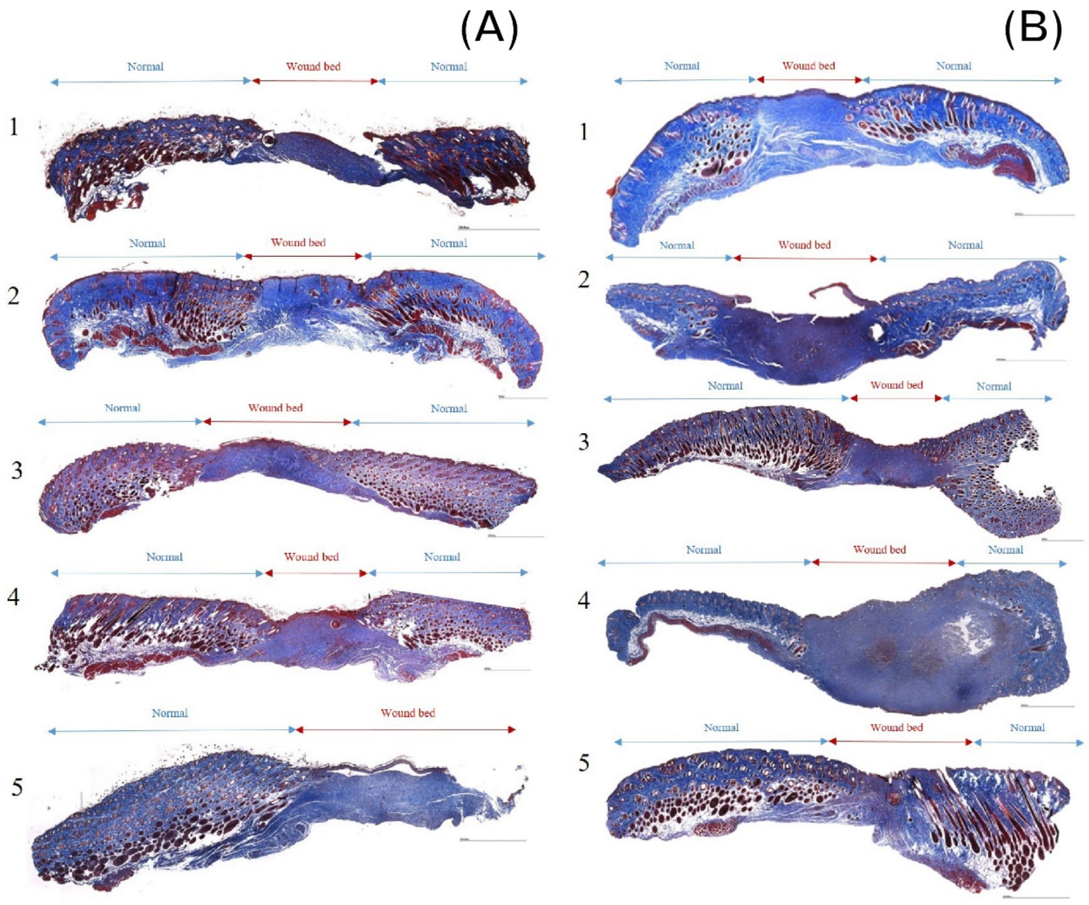

Figure S1. Pathological analysis of Masson trichrome staining of (A) regenerated tissue harvested from negative control group, (B) regenerated tissue harvested from urchin-like Ag–Au bimetallic nonwoven mat-treated group, which shows dense collagen fiber formation in the wound bed. The inset scale bar represents 100 μm. N=5 for each group.
